# Psychometric Properties of the Traditional Chinese Version of the Interprofessional Collaborative Competency Attainment Survey

**DOI:** 10.1002/nur.70037

**Published:** 2025-12-08

**Authors:** Yawen Lee, Chihlung Chiang, Yungsung Wen, Chinyi Huang, Hsintzu Li, Chihhao Lin

**Affiliations:** ^1^ Department of Nursing Changhua Christian Hospital Changhua Taiwan; ^2^ Graduate Institute of Clinical Nursing, College of Medicine National Chung Hsing University Taichung Taiwan; ^3^ Center for Faculty Development Changhua Christian Hospital Changhua Taiwan; ^4^ Department of Otorhinolaryngology‐Head and Neck Surgery Changhua Christian Hospital Changhua Taiwan; ^5^ Medical Planning Center Changhua Christian Hospital Changhua Taiwan; ^6^ Department of Big Data and Digital Promotion Center Changhua Christian Hospital Changhua Taiwan

**Keywords:** healthcare professionals, hierarchical context, interprofessional education, nursing, psychometric validation

## Abstract

This study aimed to develop and validate the Traditional Chinese version of the Interprofessional Collaborative Competency Attainment Survey (TC‐ICCAS), a culturally sensitive tool for assessing interprofessional education (IPE) competencies in Taiwan's hierarchical healthcare system. Conducted from 2022 to 2023 at a tertiary medical center and guided by the COSMIN framework, this psychometric validation study involved 324 early‐career healthcare professionals. A three‐phase process was employed: (1) translation and cultural adaptation with expert review; (2) pilot testing with 30 professionals to ensure cultural relevance; and (3) field testing using post‐IPE program surveys. Confirmatory factor analysis (CFA) of the field test data confirmed the instrument's six‐domain structure (communication, collaboration, roles and responsibilities, patient‐centered care, conflict management, and team functioning) and established its construct validity. All subscales demonstrated high internal consistency (Cronbach's *α* = 0.86–0.93). Notably, the limited discriminant validity of the “roles and responsibilities” domain was interpreted not as a flaw, but as evidence of the tool's cultural sensitivity, reflecting the authentic role overlap common in Confucian‐influenced contexts. The TC‐ICCAS provides a reliable and valid tool for evaluating IPE outcomes, filling a critical gap in culturally appropriate instruments for hierarchical settings. Its use can enhance interprofessional training and holds the potential to improve teamwork and patient safety in Taiwan and similar Asian healthcare contexts. No patient or public contribution was included as the study focused on professional competencies.

## 1 Introduction

Effective patient‐centered care relies on seamless interprofessional collaboration (IPC) among diverse healthcare teams, with interprofessional education (IPE) as a cornerstone for developing essential teamwork skills (Institute of Medicine US Committee on the Health Professions Education Summit 2003; Saragih et al. 2024). IPE fosters mutual understanding across disciplines, enhancing competencies critical for safe, holistic clinical practice (Reeves et al. 2011). Robust IPC reduces medical errors and improves patient safety through cohesive team dynamics (Hammick et al. 2007). However, evaluating IPE outcomes is challenging due to complex professional interactions, cultural influences, and limited longitudinal evidence, underscoring the need for reliable, culturally sensitive assessment tools, particularly in Taiwan's hierarchical healthcare system (Anna et al. 2024). Since IPE's introduction in Taiwan in 2007, it has become integral to hospital accreditation, with strategies like simulations strengthening IPC competencies (Lin et al. 2021). Yet, barriers persist, including small, single‐site studies and instruments like the Taiwanese Collaborative Practice Assessment Tool, which, despite strong reliability, focus primarily on nursing and lack retrospective designs (Ho et al. 2023; Huang et al. 2023). Taiwan's Confucian‐influenced culture, emphasizing indirect communication and deference to authority, often leads to understated self‐assessments with Western tools, necessitating a localized instrument (H. W. Chen et al. 2022). Widely used self‐report tools, such as the Readiness for Interprofessional Learning Scale (RIPLS) and Interprofessional Collaborative Competency Attainment Survey (ICCAS), face limitations in theoretical grounding and cross‐cultural adaptability (Allvin et al. 2024; Ganotice and Chan 2018). RIPLS lacks a robust theoretical foundation, while ICCAS's use of exploratory factor analysis (EFA) without confirmatory factor analysis (CFA) weakens its structural validity (Archibald et al. 2014; Parsell and Bligh 1999).

### 1.1 Conceptual Framework

This study integrates core interprofessional practice competencies to address these gaps, aligning with global standards and Taiwan's cultural context. Anchored in the Canadian Interprofessional Health Collaborative (CIHC) framework, it emphasizes six domains: communication, collaboration, roles and responsibilities, patient‐centered care, conflict management, and team functioning (Schmitz et al. 2017). The Interprofessional Education Collaborative (IPEC) framework complements CIHC by providing practical guidance for clinical education (Schmitz et al. 2017). This combined framework supports the development and validation of the Traditional Chinese version of ICCAS (TC‐ICCAS), tailored to Taiwan's hierarchical healthcare system.

### 1.2 Aims of This Study

This study aims to develop and validate TC‐ICCAS to assess six core IPC competencies, accounting for Taiwan's conservative self‐assessment tendencies (MacDonald et al. 2010; Tsai et al. 2013). Despite ICCAS's validation in diverse settings, its Western‐centric design and lack of CFA limit its applicability in Chinese‐speaking regions (Lunde et al. 2021; Mertens et al. 2025). The objectives are to: (1) create a culturally sensitive IPE assessment tool for Taiwan's hierarchical context, (2) enhance ICCAS's psychometric rigor using CFA, and (3) explore TC‐ICCAS's potential to improve IPC practices, reducing medical errors and enhancing patient safety. TC‐ICCAS seeks to advance IPE assessment in Asia and contribute to global standardization of interprofessional competency evaluation.

## 2 Methods

### 2.1 Study Design

This psychometric instrument development study used a descriptive correlational design to validate the TC‐ICCAS. Conducted from 2022 to 2023, this study employed a confirmatory approach to validate the established factor structure of the ICCAS, a method that builds upon the exploratory approaches used in prior validations (Archibald et al. 2014; Schmitz et al. 2017). The study adhered to the Consensus‐Based Standards for the Selection of Health Measurement Instruments (COSMIN) guidelines to ensure methodological rigor (Mokkink et al. 2024). As the focus was on quantitative adaptation and psychometric testing, qualitative COSMIN items (e.g., interviews) were inapplicable. The COSMIN Risk of Bias Checklist assessed methodological quality (see Supporting Information S1: Table A1).

### 2.2 Setting and Participants

Data were collected at a tertiary medical center in central Taiwan, leveraging its diverse interprofessional workforce (nurses, physicians, pharmacists, therapists). Participants were early‐career post‐graduate year healthcare professionals (employed 2017–2021) trained in a standardized IPE curriculum. Probationary staff and pilot test participants were excluded to minimize bias. Purposive sampling targeted 10–15 participants per item, per factor analysis guidelines (Pett et al. 2003). Of 706 invited professionals, 324 completed the survey (46% response rate), including 175 nurses (54%), 64 physicians (20%), 30 pharmacists (9%), 38 therapists (12%), and 17 others (5%). Nonresponse bias analysis showed no significant differences in age, experience, gender, education, or profession between responders and nonresponders, confirming sample representativeness.

### 2.3 IPE Curriculum Design

The Interprofessional Education and Practice Program (CCH‐IPEPP) at the study hospital was designed to strengthen post‐graduate year trainees' teamwork skills. Based on Kolb's (1984) experiential learning theory and aligned with CIHC and IPEC core competencies, it featured a multiphase, iterative approach distinct from single‐session programs. The curriculum included eight bimonthly modules blending IPE (scenario‐based simulations) with IPP (using “Briefing‐Huddle‐Debriefing” and TeamSTEPPS tools (Makary et al. 2007; American Heart Association 2023). Participants were required to complete at least one module from this program.

Centered on IPEC's four core domains—Values/ethics for interprofessional practice, roles/responsibilities, interprofessional communication, and teams and teamwork—and its 39 sub‐competencies, the program employed the “World Café” method as its primary strategy (Pluck 2025). Through structured small‐group discussions, participants first explored shared competencies in interprofessional teams, then reflected on discipline‐specific applications in intraprofessional groups.

This process enabled trainees to cocreate authentic, locally relevant teaching scenarios that integrated key IPE competencies. Prioritized sub‐competencies included:

- Communication: Actively listening to and encouraging ideas from team members.
- Collaboration: Working with stakeholders, including professionals and patients, to enhance prevention and health services.
- Roles and responsibilities: Recognizing one's own limits in skills, knowledge, and abilities.
- Patient‐centered care: Engaging professionals in patient‐focused problem‐solving.
- Conflict management: Identifying sources of conflict and engaging in constructive negotiation to resolve disagreements.
- Team functioning: Leveraging members' unique abilities to optimize care.

This trainee‐driven scenario development served as a contextual teaching tool while ensuring alignment with TC‐ICCAS‐assessed competencies.

### 2.4 Ethical Considerations

The study hospital's institutional review board approved the study (IRB No. 220116). Participants provided electronic informed consent, with assurances of anonymity, confidentiality, and the right to withdraw. Data were stored in an encrypted database, accessible only to researchers, and scheduled for destruction 1 year post‐study, per local regulations.

### 2.5 Procedure and Data Collection

Data collection (May 2022 to April 2023) followed a three‐phase process adapted from Lee et al. (2014): translation, pilot testing, and field testing.

#### 2.5.1 Translation and Cultural Adaptation

With permission from ICCAS authors, translation followed Beaton et al. (2000) guidelines. Two native Chinese‐speaking translators with medical education experience independently translated ICCAS into Traditional Chinese, emphasizing conceptual equivalence. Two bilingual English speakers back‐translated, and a consensus meeting resolved discrepancies. A six‐expert panel (two physicians, three nursing instructors, one pharmacist) confirmed content validity (content validity index [CVI] = 0.915; Davis 1992), adjusting for Taiwan's hierarchical norms.

#### 2.5.2 Pilot Testing

Thirty healthcare professionals (nursing, pharmacy, therapy; 10% of field sample; Lackey and Wingate 1998) completed the 20‐item TC‐ICCAS, rating semantic clarity (1 = very unclear, 10 = very clear) and suggesting content additions. Test−retest reliability was assessed over 2 weeks (100% retention; no intervening interventions).

The feedback from this phase led to minor wording adjustments to enhance the instrument's clarity. Crucially, the combined results of the expert panel review and the pilot test confirmed that the adapted items were culturally sensitive. The items were deemed appropriate for Taiwan's hierarchical context, effectively capturing the nuances of indirect communication styles, professional role overlaps, and deference to authority that are common in Confucian‐influenced healthcare settings.

#### 2.5.3 Field Testing

From November 2022 to April 2023, 324 participants completed an online survey after briefing and consent. The survey included demographics, the 20‐item TC‐ICCAS assessing post‐IPE competencies, and an open‐ended question on training experiences. A retrospective pre‐post design was avoided due to recall bias risks in CCH‐IPEPP's multiphase structure; instead, TC‐ICCAS focused on post‐training competency gains, tailored to cultural norms. Responses were anonymized and aggregated.

### 2.6 Instrument

The TC‐ICCAS is a 20‐item instrument adapted from the original ICCAS developed by Schmitz et al. (2017). It uses a 5‐point Likert‐type scale for all items (1 = *poor* to 5 = *excellent*). Two primary modifications were made to the original instrument to ensure its suitability for the Taiwanese context.

First, departing from the original's retrospective pre‐post design, the TC‐ICCAS employed a post‐training assessment approach. This was done to better align with the study site's ongoing curriculum structure and to minimize potential recall bias.

Second, while the six original CIHC‐aligned domains were retained to ensure theoretical consistency and allow for cross‐cultural comparison, the item content underwent significant cultural adaptation. These domains include communication (5 items), collaboration (3 items), roles and responsibilities (4 items), patient‐centered care (3 items), conflict management (3 items), and team functioning (2 items).

Specific wording adjustments were made to ensure the items accurately reflected the nuances of Taiwan's hierarchical, Confucian‐influenced clinical settings, such as indirect communication styles, professional role overlaps, and deference to authority. For instance, an item in the “Roles & Responsibilities” domain, “Be accountable for my contributions to the IP team,” was initially translated using a strong verb “prove.” However, after expert consensus, this was revised to a more neutral, duty‐focused phrase “take responsibility for,” which better reflects the cultural context of accountability without assertive overtones.

### 2.7 Data Analysis

All quantitative analyses were conducted using IBM SPSS Statistics 29 and AMOS 29 under the supervision of a consulting statistician. Descriptive statistics were first used to summarize the sample's characteristics and item responses.

The instrument's psychometric properties were evaluated primarily through CFA. CFA was selected over EFA as the TC‐ICCAS is based on the well‐established theoretical framework of the original ICCAS. As a theory‐driven approach, CFA provides robust evidence for construct validity by testing how well the observed data fit a hypothesized factor structure (Brown 2015; K. Y. Chen 2023). It also supports domain‐specific reliability by confirming that items consistently measure their intended constructs.

The CFA procedure followed a five‐step process as recommended by Brown (2015) and K. Y. Chen (2023). This involved: (1) screening for data normality; (2) model estimation to derive standardized regression weights; (3) assessing model fit against established criteria (e.g., *χ*²/df < 5, SRMR < 0.08, RMSEA < 0.08, CFI > 0.90; Hu and Bentler 1999; Hooper et al. 2008); (4) evaluating convergent validity using factor loadings, composite reliability (CR), and average variance extracted (AVE); and (5) assessing discriminant validity by comparing each domain's AVE to its inter‐domain correlations.

**Figure 1:**
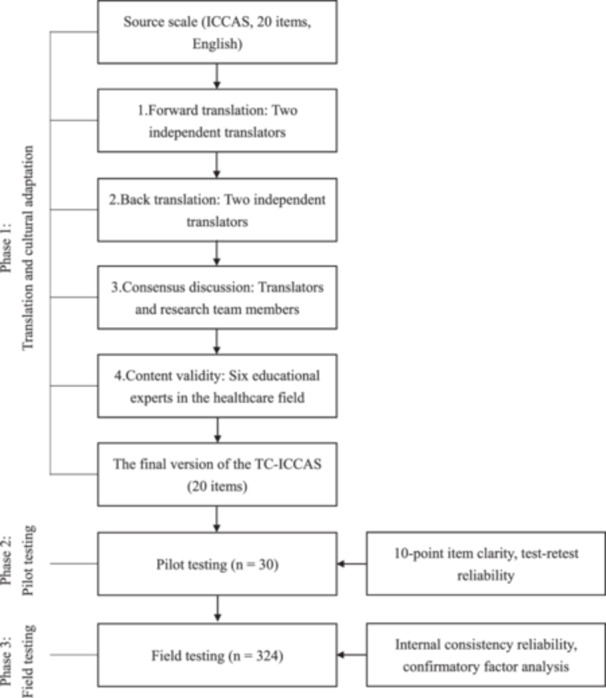
Phases of the investigation.

**Figure 2:**
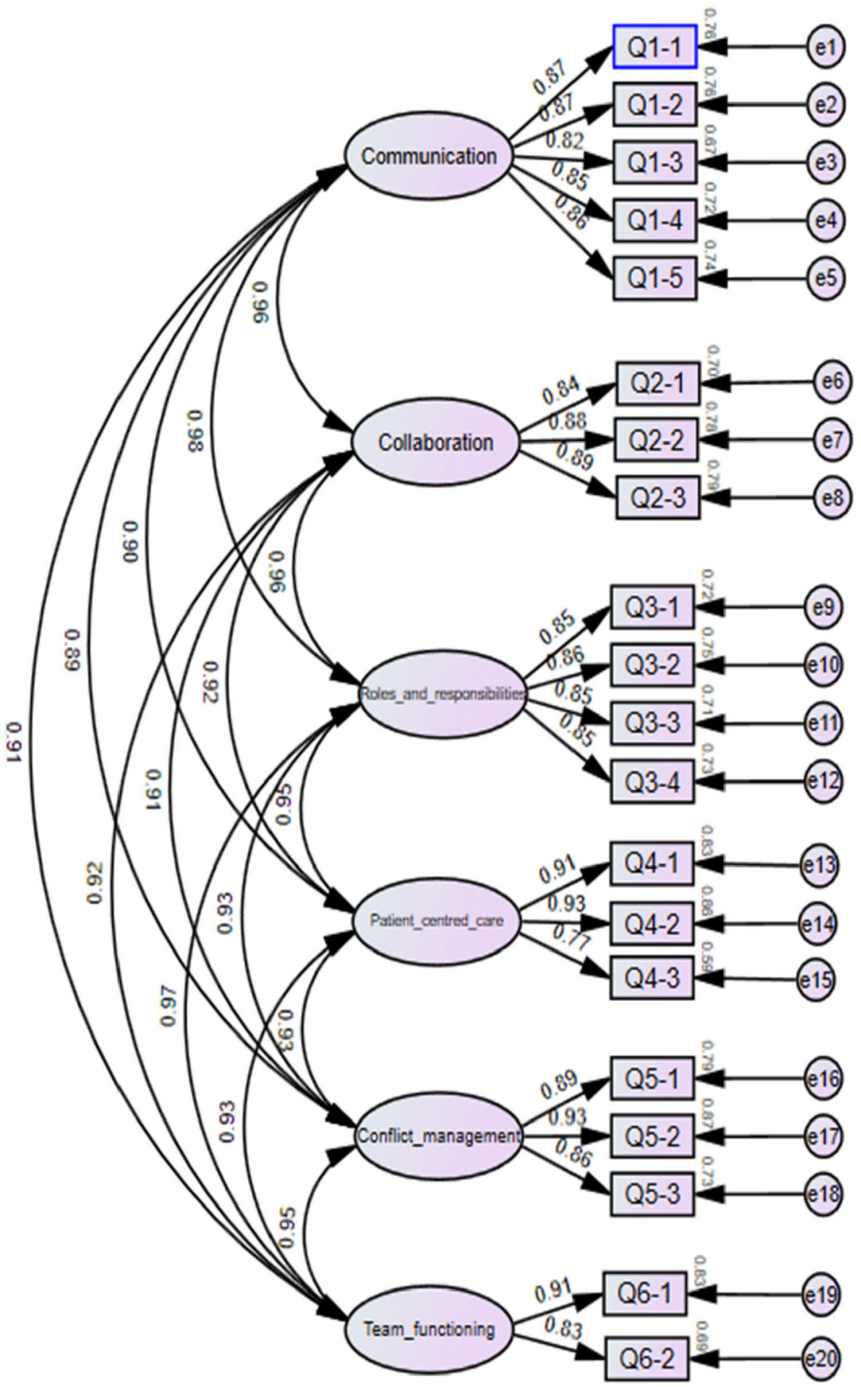
CFA diagram of TC‐ICCAS.

Following the CFA, reliability was further assessed. Internal consistency was evaluated using Cronbach's *α* for each subscale, while test−retest stability was measured with intraclass correlation coefficients (ICC) (Koo and Li 2016). Finally, to complement the quantitative findings, a qualitative content analysis was performed on the open‐ended responses to identify emergent themes (Figure 1, 2).

## 3 Results

### 3.1 Sample Characteristics

Field testing included 324 early‐career healthcare professionals (mean age = 27.4 years, SD = 2.8, range = 23.3–44.3; mean experience = 3.7 years, SD = 1.6, range = 0.9–5.8). Most were female (83.6%, *n* = 271) and held bachelor's degrees or higher (98.8%, *n* = 320). The sample comprised 175 nurses (54.0%), 64 physicians (19.8%), 30 pharmacists (9.3%), 38 therapists (11.7%), and 17 other professionals (5.2%), reflecting the IPE curriculum's diversity (Table 1).

**Table 1:** Characteristics of respondents (*n* = 324).

Characteristic	Mean (±SD)	Range	*n* (%)
Age (years)	27.4 (±2.8)	23.3–44.3	
Experience (years)	3.7 (±1.6)	0.9–5.8	
Gender			
Male			53 (16.4)
Female			271 (83.6)
Education			
Bachelor's or higher			320 (98.8)
Below bachelor's			4 (1.2)
Profession			
Nurses			175 (54.0)
Physicians			64 (19.8)
Pharmacists			30 (9.3)
Therapists			38 (11.7)
Others			17 (5.2)

### 3.2 Content Validity and Pilot Testing

The content validity of the TC‐ICCAS was established by a six‐member expert panel, yielding a CVI of 0.915, which exceeded the recommended threshold of 0.80 (Davis 1992). Following this, a pilot study was conducted with 30 healthcare professionals. Participants rated the semantic clarity of the items, with mean scores ranging from 8.20 to 8.93 on a 10‐point scale. Based on open‐ended feedback, minor wording adjustments were subsequently made to enhance clarity. The reliability of the pilot instrument was also assessed. Test−retest stability: Assessed over a 2‐week period with 100% participant retention, the instrument demonstrated acceptable stability with an ICC of 0.67 (95% CI [0.41, 0.83]).

### 3.3 Field Testing Results

#### 3.3.1 Construct Validity

CFA, following Brown (2015) and K. Y. Chen (2023), confirmed a six‐factor structure aligned with the CIHC framework (Figure 1, 2).

##### 3.3.1.1 Data Screening

Univariate normality was met (skewness = −0.47 to −0.24; kurtosis = −0.57 to 0.36; Supporting Information S1: Table A2). There was no evidence to reject the assumption of multivariate normality (Mardia's coefficient = 369.25, threshold = 440).

##### 3.3.1.2 Model Estimation

Standardized regression weights ranged from 0.77 to 0.93, with standard errors (0.13–0.16), squared multiple correlations (0.59–0.87), and error variances (0.06–0.23; Supporting Information S1: Table A2).

##### 3.3.1.3 Model Fit

The six‐factor structure of the TC‐ICCAS was evaluated using absolute, incremental, and parsimony fit indices (Table 2). Overall, the model demonstrated an acceptable to good fit. Absolute fit indices: The model reproduced the observed data well, with *χ*²/df = 3.35, RMR = 0.013, and SRMR = 0.026 meeting good‐fit criteria. RMSEA was 0.08, indicating fair fit. Incremental fit indices: All indices exceeded the 0.90 threshold, suggesting good fit (NFI = 0.93, NNFI/TLI = 0.94, CFI = 0.95, RFI = 0.92, IFI = 0.95). Parsimony fit indices: Both PNFI = 0.76 and PGFI = 0.64 surpassed the 0.50 criterion, supporting adequate parsimony.

**Table 2:** Model fit indices for TC‐ICCAS.

Fit indices	Criteria/result
Absolute fit indices	
*χ*²/df	< 5/3.35
RMR	< 0.08/0.01
SRMR	< 0.08/0.03
RMSEA	< 0.08/0.08 (90% CI = 0.06–0.09)
Incremental fit indices	
NFI	> 0.90/0.93
NNFI	> 0.90/0.94
CFI	> 0.90/0.95
RFI	> 0.90/0.92
IFI	> 0.90/0.95
Parsimony fit indices	
PNFI	> 0.50/0.76
PGFI	> 0.50/0.64

*Note:* Overall, the indices support an acceptable to good model fit. The RMSEA value suggests a fair fit, and its 90% confidence interval indicates a good fit is not supported. All estimated model parameters were statistically significant (*p* < 0.05).

Taken together, these results supported the six‐factor model. Most indices indicated good fit, though the 90% confidence interval for RMSEA (0.06–0.09) suggested that a good fit was not achieved (Hu and Bentler 1999; MacCallum et al. 1996).

##### 3.3.1.4 Convergent Validity

All domains showed strong convergent validity, with factor loadings (0.77–0.93), CR (0.87–0.93), and AVE (0.73–0.80), exceeding thresholds (0.50, 0.70, 0.50, respectively; Table 3).

**Table 3:** Confirmatory factor analysis results for TC‐ICCAS domains.

Domain	Items	SFL range	CR	AVE	Cronbach's *α*
Communication	5	0.82–0.87*	0.93	0.73	0.93
Collaboration	3	0.84–0.89*	0.90	0.76	0.90
Roles & responsibilities	4	0.85–0.86*	0.91	0.73	0.91
Patient‐centered care	3	0.77–0.93*	0.90	0.76	0.89
Conflict management	3	0.86–0.93*	0.92	0.80	0.92
Team functioning	2	0.83–0.91*	0.87	0.76	0.86

*Note:* Cronbach's *α* (> 0.70). All SFLs significant at *p* < 0.05. Additional parameters (M, SD, SE, SMC, EV) are in Supporting Information S1: Table A2.

Abbreviations: AVE = average variance extracted (> 0.50), CR = composite reliability (> 0.70), SFL = standardized factor loading (> 0.50).

##### 3.3.1.5 Discriminant Validity

Patient‐centered care, conflict management, and team functioning domains were fully distinct (square root of AVE > all inter‐domain correlations). Communication and collaboration showed moderate distinctiveness (square root of AVE > ~60% of correlations). Roles and responsibilities had limited discriminant validity (square root of AVE < all inter‐domain correlations; Table 4).

**Table 4:** Discriminant validity of TC‐ICCAS domains.

	A	B	C	D	E	F
A Communication	0.85					
B Collaboration	0.88*	0.87				
C Roles and responsibilities	0.91*	0.88*	0.85			
D Patient‐centered care	0.83*	0.85*	0.87*	0.87		
E Conflict management	0.83*	0.83*	0.85*	0.86*	0.89	
F Team functioning	0.81*	0.81*	0.86*	0.83*	0.85*	0.87

*Note:* Diagonal values are the square root of AVE; off‐diagonal values are inter‐domain correlations.

* *p* < 0.05.

#### 3.3.2 Internal Consistency

Once the six‐factor structure was confirmed via CFA, the internal consistency of each unidimensional subscale was evaluated using Cronbach's *α*. The field test (*n* = 324) revealed high internal consistency across all six domains of the TC‐ICCAS: communication, collaboration, roles and responsibilities, patient‐centered care, conflict management, and team functioning. The resulting Cronbach's *α* coefficients ranged from 0.86 to 0.93, all of which were well above the 0.70 threshold for acceptable reliability (see Table 2; Koo and Li 2016).

### 3.4 Open‐Ended Responses

Pilot feedback (*n* = 30) suggested adding items on mutual trust, diverse perspectives, and patient integration. While these valuable suggestions were not incorporated (to maintain the methodological integrity of validating the original 20‐item scale), they highlight important cultural nuances for future research. Field testing (*n* = 324) highlighted case‐based teaching's effectiveness, with participants noting its role in fostering cross‐disciplinary collaboration.

## 4 Discussion

### 4.1 Comparison of Sample and Curriculum

This study validated the TC‐ICCAS with 324 early‐career healthcare professionals in Taiwan, including nurses (54%), physicians, pharmacists, and therapists, reflecting the region's interprofessional workforce. Unlike student‐focused ICCAS validations in Norway (*n* = 1440; Lunde et al. 2021) and Minnesota (*n* = 785; Schmitz et al. 2017), this study targeted post‐graduate practitioners at a tertiary hospital, prioritizing clinical application. CCH‐IPEPP employed a multiphase, experiential learning model (Kolb 1984), contrasting with Norway's single‐session and Minnesota's 12 h interventions, which emphasized academic outcomes (Lunde et al. 2021; Schmitz et al. 2017). Integrating the “Briefing‐Huddle‐Debriefing” tool (Makary et al. 2007) and TeamSTEPPS (American Heart Association 2023), CCH‐IPEPP supported sustained skill development, aligning with the study's aim to bridge IPE to practice. CFA enhanced structural validity, addressing psychometric rigor.

### 4.2 Cultural Influences

The TC‐ICCAS was carefully adapted to ensure cultural appropriateness for Taiwan's Confucian‐influenced, hierarchical healthcare environment. While the six core IPE competency domains identified by the IPEC and embedded in the original ICCAS were retained, their application was refined to reflect Taiwan's professional practice context. For example, the roles and responsibilities domain required particular attention, as the power dynamics within hierarchical team structures can lead to a functional overlap or blurring of professional roles in practice, even when formal boundaries are clearly defined. This contextual nuance explains the more limited discriminant validity found in this domain, a pattern similarly reported in Indonesia (Dewi et al. 2024). Importantly, the adaptation process included expert review and curriculum mapping, ensuring that each item remained theoretically aligned with international standards while reflecting local practice norms. In this way, the TC‐ICCAS maintains cross‐cultural comparability yet remains sensitive to Taiwan's clinical realities.

### 4.3 Psychometric Performance

The TC‐ICCAS demonstrated strong psychometric properties. A key enhancement to the instrument's rigor was the use of CFA, which validated the proposed six‐domain structure. This approach advances beyond the original ICCAS's reliance on exploratory methods alone and provides robust evidence for the instrument's construct validity, supported by acceptable model fit indices and substantial factor loadings that were consistent with theoretical expectations (Brown 2015; Hu and Bentler 1999). Following this structural validation, reliability analyses confirmed the instrument's consistency and stability. High internal consistency was found across all six unidimensional subscales in the main field test (*n* = 324), with Cronbach's *α* coefficients ranging from 0.86 to 0.93. Furthermore, the instrument showed acceptable test–retest reliability over a 2‐week period (ICC = 0.67).

Two notable findings from the structural analysis warrant further discussion. First, although the six domains were strongly intercorrelated, this pattern is consistent with IPE theory, which posits that these competencies are conceptually interwoven and mutually reinforcing in practice (Archibald et al. 2014; Schmitz et al. 2017). The CFA results affirmed that the domains, while related, represent distinguishable facets of collaboration. Therefore, retaining the six‐domain structure is justified as it preserves theoretical consistency with the original ICCAS and facilitates international comparisons.

Second, the limited discriminant validity of the “roles and responsibilities” domain does not necessarily indicate a measurement deficiency; rather, it can be interpreted as evidence reflecting the TC‐ICCAS's cultural sensitivity. This finding likely reflects the authentic, hierarchical role dynamics and professional overlap common in Taiwan—a pattern also observed in similar contexts like Indonesia (Dewi et al. 2024). This contrasts with Western settings that often assume clearer role differentiation (Schmitz et al. 2017).

In conclusion, these findings indicate that the TC‐ICCAS is a robust instrument that precisely captures interprofessional competencies while remaining sensitive to the nuances of Taiwan's hierarchical clinical environment.

### 4.4 Implications for Practice and Policy

TC‐ICCAS strengthens IPE evaluation in Taiwan, particularly by identifying a key gap related to role clarity. Adopted by the study hospital and piloted in seven affiliates, it has improved handover processes and teamwork, potentially reducing errors, consistent with TeamSTEPPS outcomes (American Heart Association 2023). By empowering nurse‐led initiatives, TC‐ICCAS enhances care coordination, supporting sustainable quality improvements (Kolb 1984). Its integration into Taiwan's accreditation standards could standardize IPE assessment, contributing to global competency evaluation and patient safety.

### 4.5 Limitations

Several limitations of this study should be acknowledged when interpreting the findings: (1) Sample overlap in pilot testing: The use of the same sample for both the initial item review and test−retest reliability assessment may have influenced the stability estimates, potentially leading to an overestimation. (2) Absence of pre‐test data: The study's posttest‐only design did not allow for the direct measurement of competency gains over time. The findings, therefore, reflect post‐training competency levels rather than quantifiable improvement. (3) Limited generalizability: Data were collected from early‐career professionals at a single medical center. This single‐site, specific‐sample approach may limit the generalizability of the findings to other healthcare settings or to more experienced practitioners. (4) Subscale brevity: The “Team Functioning” subscale consists of only two items. While consistent with the original ICCAS, such brief scales can be psychometrically less robust and provide a narrower measurement of the construct compared to longer subscales. (5) Conceptual domain overlap: The strong intercorrelations among the six subscales suggest a degree of conceptual overlap. Although our CFA supported the six‐factor structure, future research could explore whether a more parsimonious model might sufficiently capture the core of interprofessional competency while retaining cultural sensitivity. (6) Revised and condensed version: Pilot test participants suggested adding items on mutual trust and diverse perspectives. These were not incorporated to maintain the methodological focus on validating the original 20‐item ICCAS. Nonetheless, this feedback is valuable, and future studies should consider developing an extended TC‐ICCAS that integrates these culturally relevant concepts to enhance applicability in hierarchical contexts.

## 5 Conclusion

The TC‐ICCAS has been successfully validated as a reliable and culturally sensitive tool for assessing IPE competencies within Taiwan's hierarchical healthcare context. The use of CFA supported its six‐domain structure with acceptable fit, establishing a level of psychometric rigor that advances beyond the original ICCAS and enables more meaningful cross‐cultural comparisons.

By successfully integrating cultural adaptation with methodological robustness, the TC‐ICCAS offers a practical and theoretically sound instrument to evaluate teamwork, inform curriculum design, and strengthen collaborative practice. Ultimately, the application of this tool in both clinical and educational settings holds the potential to enhance teamwork effectiveness and contribute to improved patient safety outcomes.

## Author Contributions

**Yawen Lee:** conceptualization, formal analysis, supervision, methodology, writing – original draft preparation, reviewing, and editing. **Chihlung Chiang:** project administration, formal analysis, methodology, validation, visualization. **Yungsung Wen:** conceptualization, formal analysis, supervision. **Chinyi Huang:** methodology, validation, visualization. **Hsintzu Li:** formal analysis, validation, visualization. **Chihhao Lin:** data acquisition, formal analysis, writing – original draft preparation, reviewing, and editing.

## Conflicts of Interest

The authors declare no conflicts of interest.

## Supporting information


**Table A1:** COSMIN Risk of Bias Assessment for TC‐ICCAS Development and Validation. **Table A2:** Detailed Confirmatory Factor Analysis Parameters for TC‐ICCAS Domains.

## Data Availability

Data are not publicly available due to privacy and ethical restrictions, but may be obtained from the corresponding author upon reasonable request, subject to ethical approval.
