# Supplementary material for: Psychometric Properties of the Traditional Chinese Version of the Interprofessional Collaborative Competency Attainment Survey
Source: Res Nurs Health. 2025 Dec 8;49(1):87–96. doi: 10.1002/nur.70037 (PMC12779214; doi:10.1002/nur.70037)
Supplement: Supplementary file 1 — Table A1: COSMIN Risk of Bias Assessment for TC‐ICCAS Development and Validation. Table A2: Detailed Confirmatory Factor Analysis Parameters for TC‐ICCAS Domains. [file NUR-49-87-s001.docx]

**APPENDIX A**

Table A1 COSMIN Risk of Bias Assessment for TC-ICCAS Development and Validation

| **Psychometric property** | **Item** | **Rating** | **Reason for rating** |
| --- | --- | --- | --- |
| **1. PROM Development** |  |  |  |
| *1a. Concept Elicitation Study* |  |  |  |
| 1. Target population representation | Was the study performed in a sample representing the target population? | V | Conducted in a tertiary medical center with diverse healthcare professionals, representing early-career IPE practitioners. |
| 2. Qualitative method | Was an appropriate qualitative method used to identify relevant items? | N | No concept elicitation study; relied on existing ICCAS framework. |
| 3. Skilled moderators/interviewers | Were skilled group moderators/interviewers used? | N | Not applicable; no qualitative elicitation study. |
| 4. Topic/interview guide | Were group meetings/interviews based on an appropriate guide? | N | Not applicable; no qualitative elicitation study. |
| 5. Recording and transcription | Were meetings/interviews recorded and transcribed verbatim? | N | Not applicable; no qualitative elicitation study. |
| 6. Data analysis approach | Was an appropriate approach used to analyze the data? | N | Not applicable; no qualitative elicitation study. |
| 7. Independent coding | Was at least part of the data coded independently? | N | Not applicable; no qualitative elicitation study. |
| 8. Saturation | Was data collection continued until saturation was reached? | N | Not applicable; no qualitative elicitation study. |
| 9. Quantitative sample size | For quantitative studies: Was the sample size appropriate? | V | Field testing (N = 324) exceeds 10–15 participants per item (Pett et al., 2003). |
| 10. Other flaws | Were there any other important flaws in the design or methods? | V | No major flaws identified. |
| *Total Lowest Score (1a)* |  | V | No concept elicitation study; adaptation-focused study. |
| *1b. Cognitive Interview Study or Pilot Test* |  |  |  |
| 11. Target population representation | Was the pilot study performed in a sample representing the target population? | V | Pilot (N = 30) included nursing, pharmacy, and therapy professionals. |
| 12. Comprehensibility assessment | Was comprehensibility assessed for instructions, items, response options, and recall period? | V | Semantic clarity rated on a 10-point scale; recall period not applicable. |
| 13. Final form testing | Were all items tested in their final form? | V | Final form tested; minor wording adjustments post-pilot. |
| 14. Qualitative method | Was an appropriate qualitative method used? | A | Open-ended question used; not a formal qualitative method. |
| 15. Sample size | Was each item tested in an appropriate number of patients? | V | N = 30 exceeds ≥30 threshold. |
| 16. Skilled interviewers | Were skilled interviewers used? | N | No interviews; open-ended survey question. |
| 17. Interview guide | Were interviews based on an appropriate guide? | N | Not applicable. |
| 18. Recording and transcription | Were interviews recorded and transcribed verbatim? | N | Not applicable. |
| 19. Data analysis approach | Was an appropriate approach used to analyze the data? | V | Quantitative analysis appropriate (Lee et al., 2014). |
| 20. Two researchers | Were at least two researchers involved in the analysis? | V | Two researchers confirmed. |
| 21. Problem addressing | Were comprehensibility problems appropriately addressed? | V | Minor wording adjustments; no re-testing needed. |
| 22. Other flaws | Were there any other important flaws? | V | No significant flaws in pilot design. |
| *Total Lowest Score (1b)* |  | A | Item 14 rated Adequate due to limited qualitative method. |
| *Overall Total Lowest Score (1a-1b)* |  | A | 1a = V, 1b = A; reflects robust adaptation with minor gaps. |
| **2. Content validity** |  |  |  |
| *2a. Asking Patients About Relevance* |  | N | No patient relevance assessment; expert review focus. |
| *2b. Asking Patients About Comprehensiveness* |  | N | No patient comprehensiveness study. |
| *2c. Asking Patients About Comprehensibility* |  | N | No patient comprehensibility study. |
| *2d. Asking Professionals About Relevance* |  |  |  |
| 25. Method | Was an appropriate method used to ask professionals about relevance? | V | Expert panel (n = 6) with CVI = 0.915 (Rubio et al., 2003). |
| 26. Disciplines | Were professionals from all relevant disciplines included? | V | Panel included physicians, nursing instructors, pharmacist. |
| 27. Sample size | Was each item tested in an appropriate number of professionals? | V | Six experts evaluated all items, meeting 5–10 recommendation. |
| 28-30. Analysis and flaws | (Analysis approach, researchers, flaws) | V | No flaws noted. |
| *Total Lowest Score (2d)* |  | V | All items rated Very Good. |
| *2e-f. Professionals (Comprehensiveness/Comprehensibility)* |  | N | No separate assessments beyond CVI. |
| *Overall Total Lowest Score (2a-2f)* |  | V | 2d = V; others not applicable. |
| **3. Structural validity** |  |  |  |
| 1. Factor analysis | Was exploratory or confirmatory factor analysis performed? | V | CFA performed. |
| 2. IRT/Rasch model fit | Does the chosen model fit the research question? | N | No IRT/Rasch; CTT-based CFA used. |
| 3. Sample size | Was the sample size included in the analysis adequate? | V | N = 324 exceeds 200–300 threshold. |
| 4. Other flaws | Were there any other important flaws? | V | No flaws; rigorous CFA process (Brown, 2015; Chen, 2023). |
| *Total Lowest Score* |  | V | All applicable items Very Good. |
| **4. Internal consistency** |  |  |  |
| 1. Continuous scores | Was Cronbach’s alpha or omega calculated? | V | Cronbach’s α = 0.86–0.93. |
| 2-3. Dichotomous/IRT scores | (KR-20, SE(θ)) | N | Not applicable; continuous Likert scale. |
| 4. Other flaws | Were there any other important flaws? | V | No flaws; robust assessment. |
| *Total Lowest Score* |  | V | All applicable items Very Good. |
| **5. Cross-cultural validity\measurement invariance** |  |  |  |
| 1-4. All items | (Sample similarity, analysis, sample size, flaws) | N | No cross-cultural comparison or MI analysis. |
| *Total Lowest Score* |  | N | Not applicable. |
| **6. Reliability** |  |  |  |
| 1. Patient stability | Were patients stable between repeated measurements? | V | Participants reported stable conditions. |
| 2. Time interval | Was the time interval appropriate? | V | Two-week interval appropriate. |
| 3. Measurement conditions | Were conditions similar for repeated measurements? | V | Conditions consistent; no interventions. |
| 4. ICC calculation | Was the appropriate ICC calculated? | V | ICC = 0.67, 95% CI [0.41, 0.83]. |
| 5-7. Other scores | (Kappa for dichotomous/nominal/ordinal) | N | Continuous scores only. |
| 8. Other flaws | Were there any other important flaws? | V | No significant flaws. |
| *Total Lowest Score* |  | V | All applicable items Very Good. |
| **7. Measurement error** |  |  |  |
| 1-6. All items | (Stability, interval, conditions, SEM/SDC/LoA, agreement, flaws) | N | No measurement error analysis (e.g., SEM). |
| *Total Lowest Score* |  | N | Not applicable. |
| **8. Criterion validity** |  |  |  |
| 1-3. All items | (Correlations/AUC, sensitivity/specificity, flaws) | N | No criterion validity assessed. |
| *Total Lowest Score* |  | N | Not applicable. |
| **9. Hypotheses testing for construct validity** |  |  |  |
| *9a-9b. All items* | (Convergent, discriminative validity) | N | No hypotheses testing; CFA focus. |
| *Total Lowest Score* |  | N | Not applicable. |
| **10. Responsiveness** |  |  |  |
| *10a-10d. All items* | (Criterion, construct approaches) | N | No responsiveness assessed. |
| *Total Lowest Score* |  | N | Not applicable. |

Note: Ratings: V = Very Good, A = Adequate, N = Not Applicable. Worst score counts method applied per property. IPE = interprofessional education; CVI = content validity index; CFA = confirmatory factor analysis; CTT = classical test theory; ICC = intraclass correlation coefficient; MI = measurement invariance; SEM = standard error of measurement.

Table A2 Detailed Confirmatory Factor Analysis Parameters for TC-ICCAS Domains

| Domain | Item | M | SD | SK | KU | SE | SMC | EV |
| --- | --- | --- | --- | --- | --- | --- | --- | --- |
| Communication | Q1-1 | 4.08 | .68 | -.40 | .23 | .13 | .76 | .11 |
|  | Q1-2 | 4.14 | .68 | -.36 | -.14 | .13 | .76 | .11 |
|  | Q1-3 | 4.08 | .72 | -.42 | -.12 | .15 | .67 | .17 |
|  | Q1-4 | 4.01 | .74 | -.25 | -.55 | .15 | .72 | .15 |
|  | Q1-5 | 3.99 | .74 | -.31 | -.33 | .15 | .74 | .14 |
| Collaboration | Q2-1 | 4.11 | .70 | -.36 | -.21 | .14 | .70 | .15 |
|  | Q2-2 | 4.11 | .71 | -.48 | .03 | .14 | .78 | .11 |
|  | Q2-3 | 4.16 | .70 | -.40 | -.30 | .14 | .79 | .10 |
| Roles and responsibilities | Q3-1 | 3.98 | .75 | -.45 | -.01 | .15 | .72 | .16 |
|  | Q3-2 | 4.07 | .71 | -.30 | -.38 | .14 | .75 | .13 |
|  | Q3-3 | 4.15 | .67 | -.24 | -.57 | .14 | .72 | .13 |
|  | Q3-4 | 4.14 | .68 | -.30 | -.39 | .14 | .73 | .13 |
| Patient-centered care | Q4-1 | 4.15 | .68 | -.30 | -.38 | .13 | .83 | .08 |
|  | Q4-2 | 4.17 | .70 | -.41 | -.32 | .13 | .86 | .07 |
|  | Q4-3 | 4.01 | .75 | -.42 | .15 | .16 | .59 | .23 |
| Conflict management | Q5-1 | 4.11 | .71 | -.43 | -.06 | .14 | .79 | .10 |
|  | Q5-2 | 4.16 | .69 | -.33 | -.46 | .13 | .87 | .06 |
|  | Q5-3 | 4.19 | .70 | -.39 | -.54 | .14 | .73 | .13 |
| Team functioning | Q6-1 | 4.08 | .68 | -.27 | -.27 | .13 | .83 | .08 |
|  | Q6-2 | 4.01 | .74 | -.47 | .36 | .15 | .69 | .17 |

Note: M = mean; SD = standard deviation; SE = standard error; SMC = squared multiple correlation; EV = error variance. Data from field testing (N = 324). All values rounded to two decimal places.
